# Goodpasture's Syndrome and Silica: A Case Report and Literature Review

**DOI:** 10.1155/2010/426970

**Published:** 2010-09-20

**Authors:** James Dahlgren, Marla Wardenburg, Trevor Peckham

**Affiliations:** ^1^UCLA School of Medicine-Occupational Medicine, 2811 Wilshire Boulevard, Suite 510, Santa Monica, CA 90403, USA; ^2^James Dahlgren Medical, 2811 Wilshire Boulevard, Suite 510, Santa Monica, CA 90403, USA

## Abstract

We report a case of Goodpasture's syndrome following chronic low level and an acute, high level of exposure to crystalline silica. A 38-year-old male tilesetter was admitted to the emergency room with dyspnea and respiratory failure. He reported that his symptoms had developed over the previous week after inhaling a large amount of dust while dry-sanding and sweeping a silica-based product used to fill cracks in a cement floor. Over the following days, his pulmonary function declined and he developed acute renal failure. Tests of antiglomerular basement membrane antibody were positive and renal biopsy revealed global glomerulonephritis. He was diagnosed with Goodpasture's syndrome and treated with steroids, plasmapheresis, and hemodialysis. This man had a history of childhood asthma and a remote, one pack-year history of cigarette use. He used the flooring product for seven years prior to the inciting event, however, previous jobs had utilized significantly smaller amounts. Goodpasture's syndrome and other autoimmune diseases have been reported in association with silica exposure. The acute onset following high level silica exposure in this previously healthy man, suggest that clinicians should investigate silica exposure as a causal factor in cases of Goodpasture's syndrome.

## 1. Introduction

Goodpasture's Syndrome is a rare autoimmune disease characterized by pulmonary hemorrhage and glomerulonephritis. Clinical features of Goodpasture's Syndrome include rapidly progressive renal failure and chest radiographs show localized or diffuse alveolar shadowing. Renal biopsy is needed to confirm the diagnosis and reveals a crescentic glomerulonephritis and fibrinoid necrosis. In addition, diagnostic criteria include a positive anti-glomerular basement membrane (GBM) antibody. Treatment for Goodpasture's Syndrome includes steroids, hemodialysis, and plasmapheresis. Disease progression can be halted with medical intervention; however, renal and pulmonary damage can be permanent. 

Silica, also known as silicon dioxide, is a compound commonly found in nature and is widely used. Silica is a principal component in many products, including glass, concrete, ceramics, food additives, insulation, concrete construction, foundry castings, sandblasting, and paint, to name a few. Many workers in the US are at risk of occupational exposure to respirable crystalline silica, especially jobs in industries such as construction, abrasive blasting, foundry work, stonecutting, rock drilling, quarry work, and tunneling. Occupational exposures to respirable crystalline silica are associated with the development of silicosis, a progressive fibrosis in the lung. These exposures have also been known to cause autoimmune diseases such as scleroderma, rheumatoid arthritis, and glomerulonephritis [1]. Crystalline silica is classified as a Group 1 carcinogen by the International Agency for Research on Cancer. We describe here a 38-year-old man who developed Goodpasture's Syndrome after chronic low level and an acute, high level exposure to crystalline silica.

## 2. Case Presentation

A 38-year-old man presented to the emergency room complaining of cough, dyspnea, and bloody sputum. When questioned, he stated he began to feel ill about one week earlier, after inhaling a large amount of dust during his work as a tilesetter. He reported that he had spent a three-day period (8–10 days prior to presentation) sanding and dry-sweeping a floor patching compound used to fill cracks in the cement floor. He had used this same material on many smaller jobs, but this job utilized a much larger amount of the patching material than he had used previously. He was exposed to clouds of visible dust during the mixing, sanding, and sweeping of the material. The compound contained 1% to 5% crystalline quartz silica. It was also noted that the product was highly alkaline (pH = 12). He experienced mucosal irritation while working, and had to go outside 3-4 additional times each workday to spit out dust and to “catch his breath.” He felt ill each night after work. After the third day of exposure he felt so poorly that he did not return to work. Over the following weekend, his symptoms progressed to chills and fever and he thought he had the flu. He went to walk-in clinic and was given antibiotics and an inhaler. His pulmonary problems worsened that evening, prompting him to present to the emergency room from where he was admitted to the hospital.

The patient reported a history of childhood asthma and a remote, one pack-year history of cigarette use. Physical exam at presentation was remarkable for diffuse crackles bilaterally and expiratory wheezes in all lung fields. After two treatments with nebulized albuterol and Atrovent the patient's blood gas showed a pH of 7.45, PCO2 of 36, and PO2 of 56 on room air. Chemistry panel, CPK, and troponin levels were normal. White cell count was 17.7 thou/cu mm, hemoglobin 15 g/dL, and hematocrit 42%. A chest X-ray revealed abnormalities in the lung suggestive of a diffuse interstitial pattern. Serial AFB tests and sputum cultures were negative for mycobacterium, pneumocystis, and streptococcus pneumoniae.

Two days after admission the patient became increasingly hypoxic and was intubated and put on a pressure ventilator. Fiberoptic bronchoscopy revealed bloody secretions, and BAL fluid showed no particulate matter or evidence of bacterial infection. Transesophageal echocardiogram showed normal LV function, EF 65%, no significant valvular abnormalities, and a trivial pericardial effusion. He did not respond to oral antibiotics and made no progress in weaning off ventilation, but rather progressed to severe respiratory acidosis, with an arterial pH < 7.13 and pCO2 > 100 mmHg one week after presentation. He subsequently became unresponsive and developed oliguria and azotemia. Urinalysis one week after presentation showed hematuria and mild proteinuria consistent with glomerulonephritic disease, as well as muddy brown casts characteristic of acute tubular necrosis. At this time the patient tested positive for the anti-GBM (glomerular basement membrane) antibody and he was diagnosed with Goodpasture's syndrome. This was confirmed by renal biopsy, which demonstrated global glomerulosclerosis, focal hematuria, and linear immunofluorescence reactions (IgG, IgM, C3) on glomerular basement membranes. An open-lung biopsy revealed multifocal interstitial fibrosis, diffuse interstitial PMNs (polymorphonuclear neutrophils), intravascular thrombi, and evidence of intra-alveolar damage; a constellation of symptoms consistent with Goodpasture's syndrome. No polarizing particles were seen, but immunofluorescence studies revealed linear alveolar septal IgG- and C3-immunoreactivity. Lung biopsy stains also conclusively ruled out fungi, bacteria, pneumocystis, and viral etiologies. 

The patient was then treated with continuous hemofiltration and dialysis, two weeks of plasmapheresis, and immunosuppressive drugs including Cytoxan and prednisone. Over the course of a three-month hospitalization the patient suffered complications including sepsis and cerebral hemorrhage, but renal function began to improve and he was gradually weaned off ventilation. Tests performed during the fourth week of his hospitalization were negative for anti-GBM antibody. At discharge, the patient was ambulatory and able to breathe on his own, but demonstrated permanent cognitive and motor impairment. Chest radiograph at this time revealed persistent bibasilar atelectasis and interstitial pulmonary edema. Subsequent outpatient pulmonary function testing demonstrated FEV-1 and FEV-1 : FVC values significantly below predicted, consistent with the patient's reported symptoms and an obstructive picture. Quarterly follow-up has shown no recurrence of Goodpasture's syndrome, and the patient ceased immunosuppressive drugs several months after his initial hospitalization. However, the patient has required episodic treatment for recurrent dyspnea and hemoptysis in the three years following his initial disease onset and treatment due to lasting obstructive pulmonary damage.

## 3. Discussion

Goodpasture's Syndrome was first described by Ernest Goodpasture in 1919 and later named after him in 1958 [2]. This disease is characterized by pulmonary hemorrhage and glomerulonephritis and is mediated by autoantibodies directed against the basement membrane in the renal glomerulus and the pulmonary alveolus. The etiology of Goodpasture's Syndrome is still unknown; however, development of the disease following crystalline silica exposure has been reported in at least three case reports [3–5]. Other exposures have also been implicated as causal factors, including hydrocarbons [6, 7], cocaine use [8], hard metal dust [9], and *D*-penicillamine [10]. The circumstances of our case, a tilesetter with a minor history of tobacco use, suggest inhalation of silica as the salient factor, although some exposure to hydrocarbons is not uncommon amongst workers in construction related jobs.

This patient started his 9-year career in flooring work as a helper and was later promoted to floor mechanic. His job duties included mixing and applying compound, leveling and sanding the floor, sweeping up excess dust and debris, and physically laying tile or carpeting. No safety equipment was ever used, including respirators or gloves. He reports using the compound regularly in the seven years leading up to his hospitalization. Details of other chemical exposures during his career as a tilesetter are unknown. Prior to working in the flooring industry, the patient briefly worked as a driver for a feed store, at a printing company, and at a glass company. He also worked for a pest control company for seven years, where he reports wearing protective equipment, including gloves and respirator during job duties.

Information reported by this case indicates an intense acute exposure to respirable silica prior to the onset of disease. There was not an industrial hygienist present to measure air levels during sanding and sweeping work at his final job, however, the patient reports that there were consistently large dust clouds present on each of the three work days and that the quantity of the floor patching product was tremendously greater than he had ever used in any job prior. The exact amount of the patching compound used at this site is unknown; however, purchasing record invoices for the job totaled over 10,000 pounds. It is likely that the case applied, sanded, and swept most, if not all, of the product during his three days at the job. He used a small fan to dry each application, but no dust controls, engineering controls, or other ventilation controls were used. Given a crystalline silica content of 1% to 5%, these factors indicate a maximum potential for inhalation of a high quantity of silica-containing dust prior to the onset of symptoms, presumably far exceeding recommended occupational limits (Table 1).

Crystalline silica is recognized as both a pneumotoxin and nephrotoxin, and has been reported to cause multiple autoimmune diseases [1]. As such, our case revealed severe target organ toxicity to the lungs, kidneys, and other target organ systems consistent with those reported in the literature. 

A multitude of epidemiological studies connect silica exposure to various known autoimmune diseases [13–20]. Frequently reported diseases include scleroderma, systemic lupus erythematosus, rheumatoid arthritis, autoimmune hemolytic anemia, and sarcoidosis [1]. Several studies of silica-exposed workers have shown increased rates of several common autoimmune diseases, indicating that studies that examine rates of only one disease may underestimate the risks posed by silica exposure [21, 22].

Consistent with its correlation to autoimmune effects, epidemiological data has implicated silica as a cause of renal disease [18, 23, 24]. A cohort study of 4,626 silica-exposed workers showed comparatively high rates of mortality from acute renal disease, chronic renal disease, and arthritis. There was an excess in incidence of end-stage renal disease, which was highest for glomerulonephritis. They found increasing end-stage renal disease incidence with increasing cumulative exposure [25]. Another study examined rates of renal disease in a remote mining area in the United Kingdom, in comparison to the nonmining areas of a clinical practice. Statistical analysis confirmed a significant excess of renal disease, especially glomerulonephritis, in the mining area. The authors propose that this excess is due to high silica exposure inherent to fluorspar mining. Of the six workers with long-term silica exposure, five had glomerulonephritis proven by biopsy and the other had signs and symptoms compatible with chronic glomerulonephritis [26]. These findings are consistent with other studies that show an increased risk between silica exposure and glomerulonephritis [14, 27, 28].

Case reports also describe the acute onset of pulmonary, renal, and autoimmune disease following silica exposure, detailing clinical manifestations similar to our case presentation [29–33]. One case series presents four patients who all developed rapidly progressive renal failure following high-intensity silica dust exposure. Histological findings in each case showed glomerular hypercellularity and sclerosis, crescents, interstitial cellular infiltrates, and tubular necrosis. Circulating immune complexes were present in three of the patients, and electron microscopy showed electron-dense glomerular basement membrane deposits in all of the patients. Additionally, all four cases presented with respiratory disease [34]. 

Simultaneous presence of lung and kidney failure following exposure to silica is not confined to Goodpasture's Syndrome. In a case-control study, the occupational histories of 16 cases with a clear diagnosis of Wegener's granulomatosis with renal involvement were compared with those of 32 matched controls. Wegener's granulomatosis is a systemic vasculitis with autoimmune and renal manifestations similar to Goodpasture's. Five cases reported exposure to silica dust resulting in an odds ratio of 5.0 (95% CI 1.4–11.6) for this autoimmune disease compared the nonexposed controls, and seven cases reporting exposure to any silicon-containing compounds yielded an odds ratio of 6.5 (95% CI 1.3–13.5). Reported exposure to lead, cadmium, chromium, welding fumes, and hydrocarbon was not associated with a significantly increased risk of Wegener's granulomatosis [35].

Available to us in the literature are three case reports (four cases) linking chronic exposure to silica directly to the development of Goodpasture's syndrome. Stratta et al., in a 2001 publication, proposed several reasons why there are few studies and examples of crystalline silica-related Goodpasture's Syndrome present in the literature. Reasons stated include limited records on patients with acute symptoms leading to death, lack of renal clinical data from the exposed workers, lack of correct diagnosis from the hospitals and medical institutions carrying out the dialysis, and relatively low occurrence to cross-reference to occupational factors [36]. 

One report describes a 46-year-old foundry worker with a history of intense exposure to respirable crystalline silica that was admitted to the hospital with dyspnea, hematuria, proteinuria, severe restrictive pulmonary function disorder, and rapidly declining renal function. Immunological tests and biopsy were positive for anti-GBM antibody and pauci-immune crescentic glomerulonephritis, and he was diagnosed with Goodpasture's syndrome and treated by hemodialysis and plasmapheresis. A workplace survey found that the exposure level of respirable crystalline silica exceeded the exposure limit, a TLV-TWA of 0.0106 mg/m^3^ which was calibrated for overtime. The authors conclude that based on his clinical presentation and industrial hygiene examination, his Goodpasture's syndrome was caused by long-term silica exposure [3]. 

Another case report discusses a 48-year-old male with a history exposure to silica and lead while employed a minimum of four years as a glazed-tile painter at a ceramic tile factory. Characterization of silica is absent from the report. This particular case was complicated by extensive lead exposure and a history of acute lead poisoning [4]. One other case report implicated crystalline silica in the development of Goodpasture's Syndrome in two mine workers with pneumoconiosis [5]. Levels of exposure are not described in either report, although job histories reveal chronic exposure over a number of years.

While studies suggest a causal relationship between silica and autoimmune disease, the cellular mechanism is not known. One theory is that when respirable silica particles are encapsulated by macrophages, fibrogenic proteins and growth factors are generated, and ultimately the immune system is activated [37–39]. Inhalation of large doses of silica has been reported to increase mobilization from the lungs into other organs including the kidney [40]. One can therefore hypothesize that once silica has mobilized into other organs, the immune system erroneously damages normal tissues these organs. Some reports propose that silica can induce multisystem disease through both activation of the immune system and a direct tissue toxic effect [34]. Furthermore, recent literature surveys suggest that silica has an adjuvant effect and nonspecifically enhances the immune response, therefore, precipitating a variety of systematic autoimmune diseases [15, 17]. A 2005 report by Brown et al. focuses on potential mechanisms by which silica can lead to the development and progression of autoimmune disease [13].

Similarly, the pathogenesis of glomerulonephritis and other renal effects in silica-exposed workers is not established. While some case reports provide evidence of an immunologic injury by immune complex formation, others point to a direct toxic effect of silica [23, 41–43]. It has also been postulated that since lung damage is found in several kidney diseases, such as Goodpasture's syndrome and Wegener's granulomatosis, then pulmonary insults may have a role in the development of some chronic renal diseases [44].

We speculate that the highly alkaline nature of the product resulted in an irritating and corrosive effect on the mucous membranes of the upper respiratory tract when inhaled. As a consequence of a mucous membrane injury, our case would have likely amplified vulnerability to the silica, increasing its absorption into the lung or submucousal tissue. 

Given the well-established health risks of silica dust and the temporality of disease progression, we conclude that our case of Goodpasture's Syndrome was caused by intense exposure to alkaline silica dust. This patient was in good health prior to his acute exposure and had no prior history of pulmonary, renal, or autoimmune disease. It is likely that the high dose of respirable silica dust caused his acute respiratory failure and initiated the progression of autoimmune disease, which ultimately led to renal failure. Following his recovery from the event, he never had any recurrence of the illness, suggesting that the inciting event was resolved. Multiple subsequent blood tests for anti-glomerular basement membrane antibody came up negative. 

Previous studies have focused on chronic rather than acute occupational exposure to silica; however, evidence suggests that the intensity of exposure rather than duration seems to be important in selectively causing autoimmune disorders [45]. Characterization of exposures during his career is poor, however, this patient reports sanding and sweeping the flooring compound on many occasions during a seven-year period leading up to his final job. Whether adequate exposure controls were in place during prior jobs is unknown. We do know, however, that safety equipment, such as respirators, were not used, and we can expect that chronic exposure to respirable silica took place. However, our case's experience may be somewhat unique, as we believe this to be the first reported case in which there was an acute, high-intensity exposure immediately preceding the onset of development of Goodpasture's Syndrome. 

Construction workers face an increased risk of silica-related disease due to their exposure while using, mixing, cutting, grinding, and sanding concrete and/or silica-based compounds. Over the past decades, techniques like dust suppression and increased use of respirators have lessened health risks to construction workers somewhat; yet, as our case demonstrates, silica exposure remains a pervasive hazard and important cause of disease in exposed persons. This report adds to the body of evidence supporting silica exposure as a causal factor in cases of Goodpasture's Syndrome.

## Competing Interests

Original funding of this clinical assessment was provided by a law firm representing the patient. The law firm was not involved with data analysis, data interpretation, or writing of the report. The corresponding author had full access to all the data in study and had final responsibility for the decision to submit the manuscript for publication.

##  Author's Contributions 

J. Dahlgren interviewed the patient, with the assistance of T. Peckham. J. Dahlgren, T. Peckham, and M. Wardenburg analyzed and interpreted the patient's medical and exposure data regarding the diagnosis of Goodpasture's Syndrome. J. Dahlgren, T. Peckham, and M. Wardenburg reviewed and summarized relevant literature and were major contributors in the writing of the manuscript. All authors read and approved the final manuscript.

## Figures and Tables

**Table 1:** Recommended exposure limits for respirable crystalline silica in occupational settings.

Agency	Description	Level
American Conference of Governmental Industrial Hygienists (AGCIH) [11]	Threshold Limit Value (TLV) Time Weighted Average (TWA)	0.025 mg/m^3^
National Institute for Occupational Safety and Health (NIOSH) [12]	Recommended Exposure Limit (REL)	0.050 mg/m^3^
